# Mapping Sustainable Development Goals 8, 9, 12, 13 and 15 through a decolonial lens: falling short of ‘transforming our world’

**DOI:** 10.1007/s11625-022-01112-3

**Published:** 2022-03-21

**Authors:** Judith E. Krauss, Andrea Jiménez Cisneros, Marina Requena-i-Mora

**Affiliations:** 1 University of Sheffield, Western Bank, Sheffield, S10 2TN UK; 2 Universitat Jaume I, Castelló, Spain

**Keywords:** Sustainable Development Goals, Equity, Decoloniality

## Abstract

The United Nations’ Sustainable Development Goals (UN SDGs) aspire to be integrated and indivisible, balance the three dimensions of sustainable development and transform our world by going beyond previously agreed language. Focusing on decoloniality and equity, we explore whether these aspirations are met in analysing five goals, their targets and indicators interlinking especially the economy–ecology spheres: SDGs 8 (economic growth), 9 (industry and innovation), 12 (sustainable production and consumption), 13 (climate action) and 15 (life on land). We examine two interconnected foci. Having mapped the connections which exist, according to official UN data, between these goals’ indicators, we examine definitions and delineations in SDGs 8, 9, 12, 13 and 15 through a decolonial lens, focusing on universality, absences and modernity–coloniality. A second step investigates the equity implications of these framings, using indicator data to illustrate abiding injustices. Our original contribution is thus retracing these connections and contradictions, their intellectual heritage and their equity implications in the detail of these five SDGs, their targets and indicators, combining the sustainable development and decolonial literatures in novel ways. We find that trade-offs, absences and justice shortcomings call into question the attainment of the SDGs’ objectives of leaving no one behind while safeguarding advances for people, planet, prosperity, peace and prosperity. We recognize the SDGs’ opportunity to rethink how we want to co-exist in this world. However, we argue that recognizing absences, trade-offs and equity shortcomings are key prerequisites to attain genuine transformations for justice and sustainability through the SDGs.

## Introduction

The United Nations’ Sustainable Development Goals (UN SDGs), famously, are ‘integrated and indivisible and balance the three dimensions of sustainable development: the economic, social and environmental’ (UN 2015a, p. 1). With our study, we seek to investigate these claims of connectedness and equilibrium. We examine five SDGs: 8 – economic growth and decent work, 9 – industry and innovation, 12 – sustainable consumption and production, 13 – climate action, and 15 – life on land. We answer and expand Le Blanc’s call to analyse the SDGs as a system: we investigate these five SDGs as a system of connections and often unacknowledged trade-offs, i.e. detrimental effects of attaining one SDG on another (Alcamo et al. 2020), through a lens of epistemic and global equity. We construct a decolonial conceptual framework focusing on universality (Bhambra 2004; Connell 2007), absences (Hall 1992; Santos 2001) and modernity–coloniality (Maldonado-Torres 2016; Quijano 2000) to trace their intellectual heritage, and identify what equity repercussions they entail. Consequently, our first research question is to what degree these SDGs’ definitions of goals, targets and indicators demonstrate universality, absences and modernity–coloniality. The second, ‘so what’, question then highlights these dynamics’ global-equity implications (Menton et al. 2020). Our original contribution is thus tracing the five SDGs’ intellectual heritage and justice implications, which adds to and combines sustainable development and decoloniality literatures in novel ways.

This article is structured as follows. The following section outlines some background on the SDGs and discusses some key flaws in the five SDGs we aim to investigate. After we have outlined our conceptual footing in the decolonial literature and developed our research questions, we explain our methods. We then review the indicators, targets and goals based on UN Statistics metadata through a decolonial lens, and illustrate these dynamics’ implications for global equity. We argue that the intellectual heritages identified call into question the attainment of the SDGs’ wider objective of ‘transforming our world’ for more equity and genuine sustainability: in fact, it leads us to question whether the SDGs’ sheer existence risks subverting substantive transformations.

## A decolonial lens: universality, absences, modernity–coloniality

Quijano (2000) coined the term coloniality of power, i.e. the notion that a specific way of understanding the world rooted in Western Europe was made globally hegemonic: this perspective privileges Western European understandings as the supreme and only ways of organizing the world, starting from capital and capitalism to dualist, heterogeneity-denying notions of civilization and race. As Quijano (1992) points out, through the coloniality of power, colonial domination has an epistemic dimension, as evidenced by the overwhelming dominance of scholarship from Global North contexts (Diptee 2014) and scholarship from non-dominant contexts and knowledges often facing varying degrees of exclusion (Moosavi 2020). This is because knowledge production is fundamentally imbricated in power (Noxolo 2017). Certain forms of knowledge were privileged through modern sciences (Santos et al. 2007), while leading to the discrimination of those whose knowledges and insights were considered as neither scientific nor relevant (Tuhiwai Smith 2012). Given that ‘the Western understanding of the world is as important as it is *partial*’ (Santos 2014, p. 164, emphasis added), we follow authors who argue that ‘there is no global social justice without global cognitive justice’ (Santos 2007, p. 63). Consequently, it is important to identify how the coloniality of power has shaped a key global governance framework, the Sustainable Development Goals. To construct a decolonial lens for epistemic and global equity, we rely on three key elements: absences, modernity–coloniality and universality.

### Absences

As Hall (1992) explains, specific understandings not only construct a topic in a certain way, but also limit alternative ways to conceive of and interpret that idea. According to Santos (2001, 2014), what does not exist or what cannot be conceptualized, with the existing knowledge systems and tools, remains absent from our own understanding of the world, requiring processes of translation and manifestation to sharpen public consciousness: ‘The sociology of absences invents or unveils whatever social and political conditions, experiments, initiatives, conceptions have been successfully suppressed by hegemonic forms of globalization; or, rather than suppressed, have not been allowed to exist, to become pronounceable as a need or an aspiration’ (Santos 2001, p. 191). The sociology of absences extends Hall’s insight on the importance of a topic’s conceptualization limiting other understandings by emphasizing that absences, as we will identify in the SDGs in terms of connections not made and injustices not addressed, deserve equal attention.

### Modernity–coloniality

As Maldonado-Torres (2016) puts it, decoloniality is rooted in turning away from modernity–coloniality, an idea which was coined by Quijano (1992, 2007) and further developed, e.g., through a modernity–coloniality research programme (Escobar 2007; Mignolo 2006). Modernity and coloniality are viewed as two sides of the same coin, despite modernity promising salvation and coloniality imposing imperial oppression, as modernity’s unfinished project carries coloniality on its shoulders (Mignolo 2006, p. 312). Consequently, certain structures of knowledge, power and governance continue Western modern-colonial imposition (Walsh 2018, p. 187). According to Grosfoguel (2011, p. 13), ‘[t]he same way as the European industrial revolution was achieved on the shoulders of the coerced forms of labor in the periphery, the new identities, rights, laws, and institutions of modernity such as nation-states, citizenship and democracy were formed in a process of colonial interaction with, and domination/exploitation of, non-Western people’.

### Universality

As Raewyn Connell (1997, 2007) argues, most of the classical texts, even though written from specific geographical locations, i.e. the metropole, claim to speak in universal terms – the texts’ and the authors’ locality must, in fact, remain tacit, as any explicit recognition would question the texts’ assumed universal applicability (2007). One example is Eurocentrism, ‘[…] an epistemic phenomenon that received its name from the territorial location of actors, languages, and institutions that managed to project as universal their own world sense and worldviews’ (Mignolo 2018, p. 194). According to Connell (2007), this claim of universality goes hand in hand with an overemphasis on problems arising in metropolitan theoretical literature, an exclusion of non-metropolitan authors and the erasure of colonial experiences. In Mbembe’s words, colonialism is portrayed ‘[…] as a normal form of social relations between human beings rather than a system of exploitation and oppression’ (2016, p. 32).

The patterns of domination enacted through universality and modernity–coloniality become manifest for instance in the modernization-theory argument that poorer regions could make progress by imitating strategies applied in Western industrialized countries (Makki 2015). Modernization theory, based on, e.g., Rostow (1960), proposed a push for a specific brand of economic development, suggesting that economies’ evolution from agrarian to industrial and then to post-industrial would equate to societal progress. In Bhambra’s (2004) words, the particular experience of Western modernization was thus transformed into a global frame for all, with all differences from the norm understood as a failure of transition. Countries which are industrialized and modern continue to be seen as more ‘developed’ and vice versa (Hickel 2019), with the ‘Third World’ seen as backward and in need of being modernized to conform to the universally beneficial, Western social, cultural, environmental and structural forms (Grosfoguel 2000; Makki 2015). With Western capitalist nations presented as the pinnacle of economic and social accomplishment, the dispossession they have inflicted along the way is erased (Larrabure 2017), while neglecting to engage with the finite availability of natural resources—arguably, this is an example of absences as explained above.

Modernization theory and cognate universalist ideas rooted in modernity–coloniality are present across diverse economy–ecology relations. Mobilizing resources to improve human wellbeing, especially for those who are disadvantaged, is vital, but only if absolute biophysical limits are not violated (Fischer-Kowalski 2019; Hickel 2020; Lim et al. 2018; Rockström et al. 2009). Nevertheless, this disregard for biophysical limits is part of an imperial way of life that elevates infinite economic growth to a shared, hegemonic ideal that may be dressed up in the language of (catch-up) ‘development’ in the Global South (Lang and Hoetmer 2018). This mode of living, practised generally by the globally wealthiest quintile, generates severe socio-ecological consequences elsewhere, i.e. an ecological debt or socio-ecological subsidy (Martinez-Alier 2002a; Requena and Brockington 2021; Rice 2009). Presenting this lifestyle as universally desirable is premised on ignoring these debts, and neglecting that environmental degradation is likely to affect non-industrialized countries, and more vulnerable populations within them, disproportionately (IPCC 2018). Similarly, there are abiding colonial understandings shaping conservation (Adams and Mulligan 2003; Martinez-Alier 2002a, b; Menton et al. 2020) such as the cult of wilderness. Focusing on beautiful landscapes and threatened species, this understanding loves the environment as pristine nature. However, it also involves setting humans apart from nature without questioning who benefits or what knowledges these boundaries are based on, often to the detriment of the vulnerable (Martinez-Alier 2002b). In all these instances, universal paradigms shaped in modernity–coloniality govern relations irrespective of their socio-ecological consequences for the disadvantaged, hampering both equity and genuinely sustainable human living.

In summary, our manuscript builds a decolonial lens with three main elements: an attention to absences, the abiding impositions of modernity–coloniality, and the supposed universal applicability of one specific set of priorities and understandings. Our original contribution is retracing in the detail of five SDGs targets and indicators how these very specific worldviews rooted in absences and modernity–coloniality are promoted as universally applicable, yet in fact codify inequity (Bhambra 2004; Madianou 2019; Quijano 2000). In essence, we argue that the SDGs, despite their holistic and integrative language, are embedded in and perpetuate problematic structures through the definitions and delineations enshrined in their goals, targets and indicators. After introducing the SDGs and our specific research questions in the next section, we explain our methods.

## Premise and promise of the SDGs

### The evolution of goals, targets and indicators

The 17 SDGs were passed by the United Nations in 2015 in the resolution ‘Transforming our world—the 2030 Agenda for Sustainable Development’ to promote people, planet, prosperity, peace and partnerships (UN 2015a). The initial impetus for drafting the Sustainable Development Goals came from Paula Caballero Gómez from Colombia’s Ministry of Foreign Affairs (Chasek and Wagner 2016). Since the Millennium Development Goals were driven by a small group of rich countries (Sen and Mukherjee 2014), the ‘The Future We Want’ Rio+20 document resolved ‘to establish an inclusive and transparent intergovernmental process on sustainable development goals that is open to all stakeholders’ (UN 2012, para 248). An Open Working Group was established to draft goals and targets (Breuer et al. 2019; Le Blanc 2015). This inclusive, consultative process aimed to chart a new path in both content and language as, in the words of the Open Working Group’s co-chair Csaba Kőrösi^1^: ‘How can you construct a vision of the future from previously agreed language?’ (quoted in Chasek and Wagner 2016, p. 409).

To measure 17 SDGs and 169 targets, the Inter-agency and Expert Group on SDG Indicators (IAEG-SDGs) was established by the UN Statistical Commission in 2015 to identify suitable indicators (Lucci and Lally 2016; Rickels et al. 2016). Defining SDG indicators is a vital step in measuring transitions towards sustainability: the SDGs’ indicators form the basis of both the high-level political forum’s regular reviews and countries’ voluntary national reviews (e.g. Ordaz 2019; UN 2018a, UN 2019a). Reviewed in what was billed as an inclusive consultation process involving public sector, civil society, academia and private sector, the initial 300 proposed indicators resulted in ca. 230 SDG indicators being presented in 2016 (Rickels et al. 2016).^2^ As of December 2019, there are 232 indicators, though some are used under several targets (UN 2019b). Hák et al. (2016) observe that the SDG indicators’ conceptual framework has underlying weaknesses in terms of theorizing carefully what is measured and how to measure it. Consequently, we answer their and Hák et al.’s (2007) call for more conceptual discussion: we investigate the definitions and delineations in our five SDGs, their targets and indicators through the above-constructed decolonial lens of epistemic and global equity.

### Investigating the SDGs through a decolonial lens: modernity, universality, absences and equity

Firstly, it is important to investigate to what extent these SDGs reproduce modernity–coloniality, universality and absences. Sen and Mukherjee (2014) had called for the post-2015 development agenda to move beyond the Millennium Development Goals-inspired issue silos by centring people’s needs. Many question whether the SDGs attained this shift given Nilsson and Costanza’s (2015) observation of continuing silos in the SDGs, with Salleh diagnosing this silo thinking as being rooted in abiding humanity/nature dualisms imposed globally through capitalism and the Eurocentric cultural domination it spreads (2016). This silo thinking, i.e. the lack of attention to crucial socio-ecological connections, could be understood as an absence (Santos 2001, 2014). Equally, the SDGs have been alleged to reproduce dominant understandings of economy and ecology (Salleh 2016; Hope 2020; Weber 2017). It has been argued that the SDGs, neglecting environmental and ecosystem concerns (Reid et al. 2017), continue current infinite-growth-fixated interpretations of sustainable development without regard for planetary limits and ecological integrity (Eisenmenger et al. 2020; Hickel 2019, 2020; Lim et al. 2018) nor global equity (Gupta and Vegelin 2016; Menton et al. 2020). We will investigate in our empirics to what extent these questions about absences, universal notions and paradigms rooted in modernity–coloniality are present in the detail of our five SDGs.

Secondly, we will investigate the equity implications of these dynamics in goals, targets and indicators. The SDGs risk perpetuating long-standing, problematic myths about poverty being the cause of environmental degradation in the Global South (Broad and Cavanagh 1993; Dunlap and York 2008). These have been repeated up to and including the Brundtland Report, which forms the basis of the sustainable development agenda (Guha and Martinez-Alier 1997). Relatedly, the SDGs embody a double-bind structure (Bateson 1988) in which two contradictory commands are imposed concurrently: ‘Live as if the environment does not matter because, otherwise, you are threatened by poverty and unemployment’ and ‘Protect nature because, otherwise, you are threatened by catastrophe and extinction’ (García and Cabrejas 1996, p. 78). Brand and Wissen (2012, 2017) coin the idea of an imperial mode of living, predicated on infinite growth and mass consumption for a privileged minority. Explicitly focused on the wealthy, this idea of an imperial mode of living criticizes the socio-ecological consequences which this approach entails globally and locally for the most vulnerable by, in Illich’s words, the rich making murderous demands on the resources of the poor (1973). The socio-ecological consequences caused elsewhere of this wealthy lifestyle are problematic from a viewpoint of right relations, i.e. living up to one’s responsibility in all relationships with other humans or the environment (Gram-Hanssen et al. 2021). In our empirics, we will thus investigate the equity implications of these five SDGs, between rich countries and the rest of the world, and locally for the disadvantaged.

## Materials and methods

We focus on five goals:

- SDG 8: ‘[p]romote sustained, inclusive and sustainable economic growth, full and productive employment and decent work for all’ (UN 2019b, p. 8),
- SDG 9: ‘[b]uild resilient infrastructure, promote inclusive and sustainable industrialization and foster innovation’ (UN 2019b, p. 9),
- SDG 12: ‘[e]nsure sustainable consumption and production patterns’ (UN 2019b, p. 12),
- SDG 13: ‘[t]ake urgent action to combat climate change and its impacts’ (UN 2019b, p. 14),
- SDG 15: ‘[p]rotect, restore and promote sustainable use of terrestrial ecosystems, sustainably manage forests, combat desertification, and halt and reverse land degradation and halt biodiversity loss’ (UN 2019b, p. 16).

We acknowledge that our analysis, by focusing on 5 out of 17 goals, does not cover the full picture, but invite further research to analyze those not covered here given limited space and the objective of in-depth analysis. There are three main reasons for selecting these five goals. Firstly, SDGs 8, 9, 12, 13 and 15 scored highly in terms of trade-offs across SDGs in Pradhan et al.’s analysis (2017) of time-series data provided by the UN Statistics Division between 1983 and 2016 on 230 SDG indicators. Secondly, these goals are at the forefront of the world’s recent emphasis on gross domestic product (GDP) growth and its consequences for the environment (Costanza et al. 2016; Sen et al. 2010), and the risks which environmental degradation entails particularly for more vulnerable populations (IPCC 2018). Finally, there has been a historical tendency of economic–ecological foci to rely heavily on constructs and biases determined by the Global North (Duffy et al. 2019; Salleh 2016; Weber 2017). We equally acknowledge that our focus on goals with high trade-off scores shapes our findings. However, given the SDGs’ claims of being indivisible, identifying these tensions is arguably all the more important.

Modelling our network analysis broadly on Le Blanc’s (2015) SDG study, our first step was mapping the degree to which goals 8, 9, 12, 13 and 15, as of 1 February 2020, acknowledge connections between and beyond themselves (UN Stats 2020).^3^ We analysed the metadata on each indicator provided by the monitoring custodian organizations via the UN Statistical Division, focusing on the specified related indicators at the end of each metadata sheet.^4^^,^^5^ Every ‘related indicator’ was counted as an official connection recognized by the UN. We collated this into a database and used Gephi 0.9.2 (Chen 2015; Cherven 2015) to create a network. Analyzing recognized connections was a precursor to identifying absences. To find unacknowledged connections, we consulted SDG metastudies conducted by ISCU/ISSC (2015), Nilsson et al. (2016), Pradhan et al. (2017), Lim et al. (2018), Kroll et al. (2019), Lusseau and Mancini (2019), Barbier and Burgess (2019), Alcamo et al. (2020) and Scharlemann et al. (2020), as well as e.g. Hickel (2019), Menton et al. (2020), Weber (2017), and Weber and Weber (2020) on specific SDGs. These five SDGs, their targets and indicators were reviewed through our above-explained decolonial lens.

In reviewing our findings in terms of equity, we grouped countries into Global North and Global South on the basis of the categorizations by the Finance Centre for South-South Cooperation (2015) and standard country area codes for statistical use.^6^ We compared and made correlations between different indicators from our selected SDGs with two key environmental indicators: CO_2_ emissions per capita and material footprint per capita (Hickel 2020) in light of a finite planet and climate change’s disproportionate impact on vulnerable populations (Figs. 2–5). We render both indicators in consumption-based terms: this means they account for international trade by adding the emissions and materials embodied in imports, including the upstream emissions and resources involved in producing and shipping imported goods, while subtracting those of exports (Wiedmann et al. 2015). This allows us to account for the fact that, in an era of globalization, high-income countries have shifted much of the extraction and production side of their consumption abroad, effectively outsourcing their ecological impact (Hickel 2020). For material footprint, Bringezu (2015) uses a planetary boundary of 50 billion tonnes per year (which human consumption currently exceeds by 82%); Hickel (2020) converts this into a per-capita level of 6.7 t per year.^7^ For CO_2_ emissions, we used the planetary boundary calculated by Hickel (2020) based on IPCC’s 2018 report, i.e. 1.7 t per person per year until 2100.^8^

## SDGs 8, 9, 12, 13 and 15: decoloniality and equity

### Overview: connections in SDGs 8, 9, 12, 13, 15

**Fig. 1:**
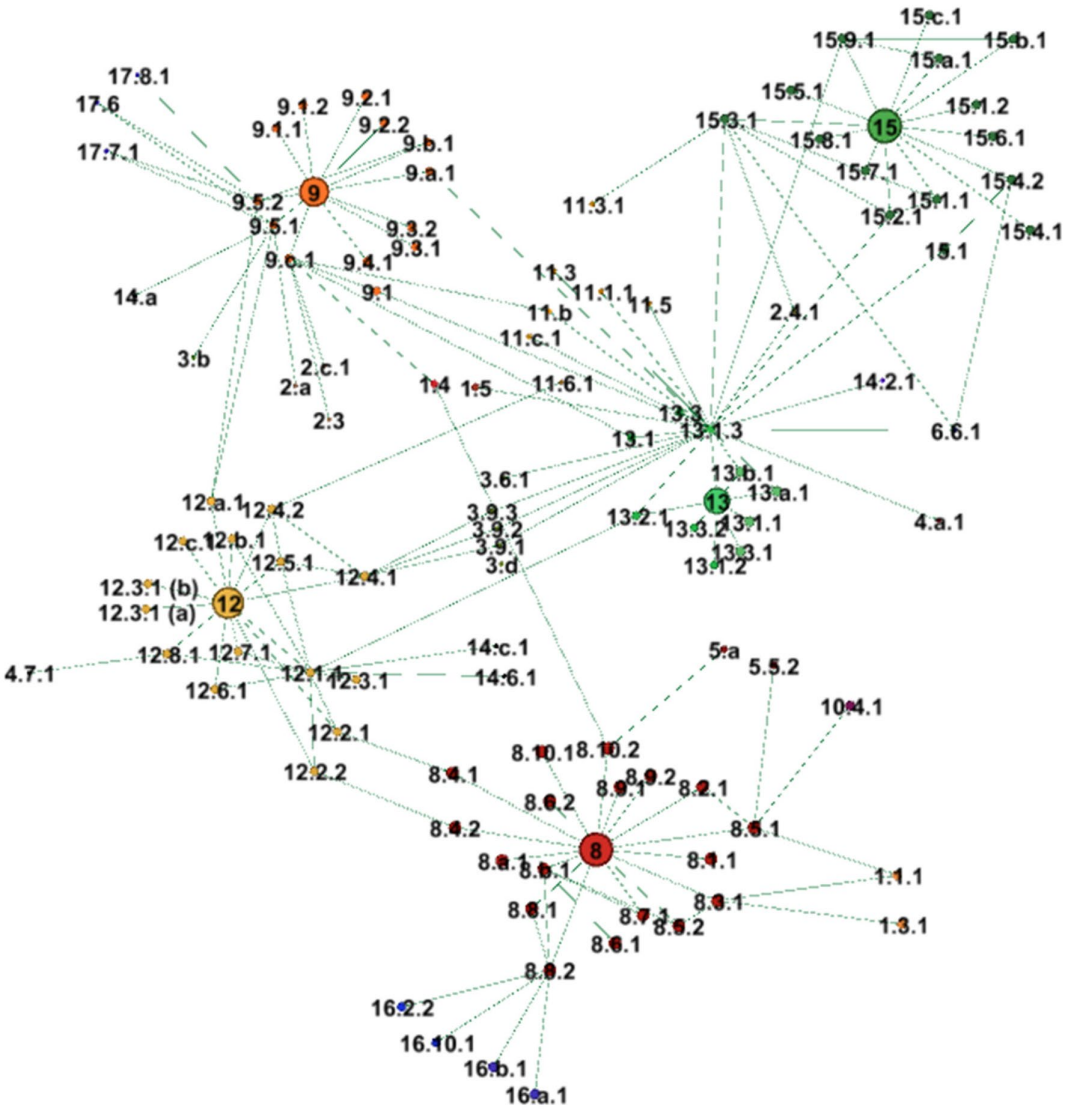
Connections of SDGs 8, 9, 12, 13 and 15 (goals, targets, indicators). Source: Authors, based on UN (2019b)

The below graph (Fig. 1) shows SDGs 8, 9, 12, 13 and 15 all as slightly larger nodes compared with their indicators, which are immediately clustered around them.^9^ The network depicts all connections, i.e. acknowledgements in UN metadata of an indicator being related to another indicator, between our five goals and to other goals and indicators.

It is apparent (Fig. 1) that acknowledged connections are fairly limited. This first impression is confirmed by in-depth analysis (Appendix 1, Appendix 2). Across the 65 indicators investigated, only 93 connections are acknowledged, i.e. under 1.5 connections per indicator on average. However, this average is skewed firstly by one indicator stating 24 connections, 13.1.3 (local government action on climate change). Secondly, as of 1 February 2020, there are numerous indicators that continue not to have UN metadata (11). Indicators without metadata often are also Tier III indicators (no data nor methodology), which are especially frequent in SDGs 12 and 13. Moreover, there are 33 indicators, i.e. half the sample, which have no acknowledged connections at all, particularly prevalent in SDGs 8 on growth (9), 9 industry (9) and 15 on life on land (10) (see Appendix 3 for more details on existing and absent connections). While we are not assuming that a greater number of officially recognized connections would automatically equate to progress, we highlight, in line with Hall (1992), that framing issues such as economic growth and natural resources as unconnected has consequences, especially in a globally applicable indicator framework aiming to ‘transform our world’. With Santos (2001, 2014), the absence of recognized connections between inherently linked issues risks missing important trade-offs, to the detriment of attaining the SDGs’ objectives as we detail further below. Equally, this lack of acknowledged connections perpetuates the risks inherent in SDG implementation currently being focused on single goals (Alcamo et al. 2020).

### Absences

We will first show in the detail of our five SDGs to what extent unacknowledged absences support Gupta and Vegelin’s (2016) suggestion of abiding silo thinking in the SDGs. Target 9.1^10^ is committed to developing resilient, sustainable infrastructure for economic development and human wellbeing with an emphasis on equitable, affordable access. However, it does not link to SDG 8, 12, 13 or 15, despite mentioning economic development, sustainable infrastructure and wellbeing; it thus ignores the extractive mechanisms required to produce infrastructure and its long-term relevance for resource consumption. Its two indicators measure firstly the proportion of a population living within 2 km of an all-season road (9.1.1), and passenger and freight volumes by mode of transport (9.1.2). The indicators thus appear to suggest that living within 2 km of an all-season road as well as high trade volumes equates to equitable access to sustainable, resilient infrastructure while safeguarding economic development and human wellbeing. While the target would be difficult to measure with any single indicator, the ones chosen seem reductive, with the absence of connections reducing the likelihood of attaining genuinely sustainable infrastructure.

A second key absence are links to ecological integrity and absolute biophysical boundaries (Eisenmenger et al. 2020; Spangenberg 2017). 8.4.1, material footprint, as an indicator is identical to 12.2.1, while 8.4.2, domestic material consumption, is the same for 12.2.2, always analysing by country, per capita and per unit of GDP. The two, which represent both consumption and production (UNEP 2017, 2018), break levels of resources consumed down to relative levels rather than measuring them against absolute planetary boundaries (Hickel 2019). Secondly, while 8.4^11^ acknowledges implicitly the role of economic growth in driving environmental degradation by highlighting the need for relative decoupling,^12^ it does not quantify nor measure necessary decoupling, never mind the ca. 7% of annual decoupling required within a hard cap of material footprint (Hickel 2019). It equally does not quantify how industrialized countries, according to the target, are to take the lead on increasing efficiency. Connections to SDG 9, industry and innovation, SDG 13, climate action, and SDG 15, life on land, are absent also from SDG 12 on sustainable consumption and production. Equally, SDG 15 does not connect to climate action, SDG 13, although the prior consultation process on SDG 15 indicators had produced suggestions to include ‘net forest emissions’ or a ‘carbon stock in woody biomass’ indicator (UN 2015b).

Maybe most questionably, there is no commitment in SDG 13, or indeed any SDGs investigated here as of 1 February 2020, to limiting overall carbon emissions (Menton et al. 2020). As Lecocq (2015) welcomes, SDG 13 acknowledges that the UN Framework Convention on Climate Change (UNFCCC) is the primary forum for negotiating global responses to climate change. However, as firm action on cutting carbon emissions has made slow progress under UNFCCC, this raises questions whether a carbon commitment in the SDGs would not be essential (Hickel 2019), especially by the Global North, e.g. to Hickel’s (2020) planetary boundary of 1.7 t per person per year, or O’Neill et al.’s (2018) 1.6 t per capita based on the Paris Agreement. Given the significant carbon repercussions of SDGs 8, 9 and 12, this seems like a key absence.

In terms of equity implications, SDG 15 has no connections to SDG 1, no poverty, SDG 8, SDG 10 on reducing inequalities, SDG 12, or SDG 13. This is particularly surprising as protected areas, on which SDG 15 relies e.g. in 15.1, 15.2 and 15.4, in their strict varieties can entail significant social and economic repercussions for local residents (Brockington and Wilkie 2015). While the mainstream, monetary-based and indicator-focused understanding of poverty is problematic in itself (Lang and Hoetmer 2018), not acknowledging any link between conservation and livelihoods is arguably worse. Perpetuating protected areas while failing to link to livelihoods cements a notion of nature conservation which often shuts out residents, recalling North American conceptualizations of national parks and constructs of colonial conservation (Brockington et al. 2008). SDG 15, both in content and indicators, thus places the focus on limiting local communities’ resource use through protected areas, rather than privileged visitors’. Moreover, the lack of acknowledgement of the role of indigenous, local and traditional knowledges in using and managing biodiversity, which was referenced in Aichi Biodiversity Target 11, but not SDG 15 (Baptiste and Martín-López 2015), is also a significant absence in terms of valuing non-dominant voices (Krauss 2022).

Overall, these lacking connections thus reproduce what Santos would call absences (2001, 2014). The lacking connections to climate action and life on land are particularly problematic as social and economic progress 2000–2016 has come at the expense of environmental SDG indicators (Barbier and Burgess 2019): the absence of connections in the official indicators thus questions to what degree these interrelations would be monitored under the SDG framework.

### Modernity–coloniality in the SDGs

**Fig. 2:**
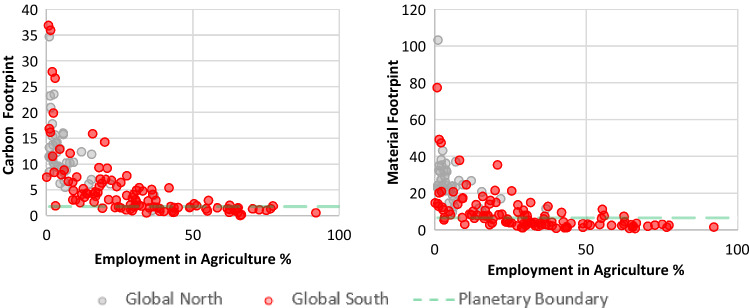
Relationship between material and carbon footprints per capita (2017) and the percentage of people employed in agriculture (2019). Source: Own figure based on EORA-MRIO (2016), United Nations Environment Programme (UNEP) (2020), World Bank (2020b), and Human Development Data (UNDP 2020)

**Fig. 3:**
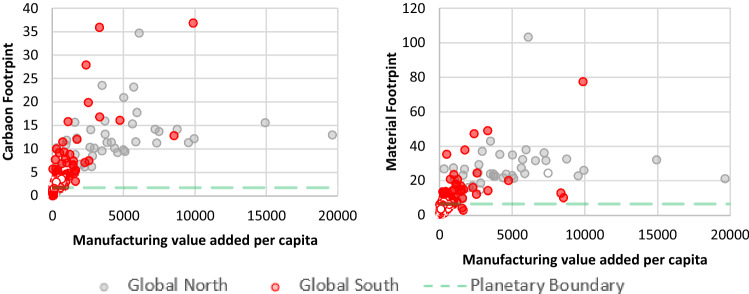
Relationship between material and footprint per capita (2017) and manufacturing value added (2016, constant US dollars 2010). Source: Own figure based on EORA-MRIO (2016), United Nations Environment Programme (UNEP) (2020), and World Bank (2020b)

The SDGs’ definitions of technology and innovation are one example of understandings rooted in modernity–coloniality. As Tukker (2015) contests for SDG 9,^13^ sustainable infrastructure, a crucial tenet of the goal, remains undefined. What implicit or explicit definitions there are advocate particular understandings of technology, innovation or sustainable development. In 9.2,^14^ industrialization is still seen as crucial, as countries should ‘significantly raise industry’s share of employment and gross domestic product’ (UN 2019b, p. 9, cf. also Esquivel 2016). This is premised on the modernization-theory assumption that industrialization and economic growth are necessary for achieving development (Hickel 2019). However, data show (cf. Fig. 2 below) that, once a country reduces their percentage of people employed in agriculture and the economy grows by becoming more industrialized, both their material and carbon footprints increase (*r* = −0.59, *p* = 0.00). A similar picture emerges when analyzing another indicator for 9.2, manufacturing value added per capita. Countries which perform well at target 9.2 – mostly from the Global North – still demonstrate resource-intensive footprints (*r* = 0.58, *p* = 0.000; Figs. 2 and 3). The juxtaposition of agricultural employment and manufacturing value added per capita with material and carbon footprints, respectively (Figs. 2 and 3), thus shows that what is being measured by SDG 9 does not advance genuinely more sustainable resource use.^15^ SDG 9 is thus not only rooted in modernity–coloniality, but privileges Global Northern (grey bubbles) countries. Global South countries (red bubbles) with higher percentages of employment in agriculture or low manufacturing value added would be seen as performing poorly on SDG 9 indicators, yet are generally the ones who have lower material and carbon footprints.

In equity terms, modernity–coloniality produces further issues. To measure Target 9.4 on retrofitting infrastructure for sustainability, indicator 9.4.1 is using CO_2_ emissions divided by unit of value added, i.e. GDP on a global scale. Firstly, overall CO_2_ emissions have not been used by SDG 13 as an indicator as of February 2020, meaning that the SDG indicator framework does not facilitate the juxtapositions with carbon footprint which we showed above. Here, carbon emissions from fuel combustion and manufacturing are used to measure relative decoupling of carbon emissions from GDP. However, carbon efficiency does not account for absolute numbers: countries performing well at carbon intensity show high CO_2_ emissions per capita. Dividing material footprint or domestic material consumption by GDP, as is also done by indicators 12.2.1, 12.2.2, 8.4.1 and 8.4.2., promotes a false perception that wealthier countries are doing more for the environment (Requena and Brockington 2021). As Martinez-Alier (2004) has pointed out, if the material intensity or carbon intensity of the economy decreases, ‘this may appear to be a good sign, and is in fact better than the contrary. GDP is of little relevance to the environment, if we may put it that way, and what matters is the absolute measure’ (2004, p. 25–26). Lastly, this does not correct that production-based analyses of CO_2_ attribute rich consumers’ carbon emissions to citizens in poorer countries to which production has been relocated (Jackson and Victor 2019). The difference between production-based and consumption-based emissions has been labelled as accumulation by displaced emissions (Isenhour and Feng 2014), given that they allow for the reproduction of affluence in one context while attributing the mitigation responsibility to another. Consumption and territory-based methods of accounting and the division by GDP thus all downplay the Global North’s responsibility for reducing environmental damage, raising significant questions of equity.

This question of who is seen as needing to make changes recurs throughout the SDGs studied, with further problematic equity implications. There is no mention of distribution of responsibility or resources in SDG 12, only one implicit reference: 12.a.1 discusses the support provided to developing countries on sustainable production and consumption technologies (12.a.1), a Tier III indicator without data and methodology. Although 9.1, 9.2, 9.4 and 9.a include words like sustainable or ‘environmentally sound technologies’, the indicators do not make any reference to equity or planetary boundaries. Instead, they measure for 9.a.1 total overseas development assistance for infrastructure, 9.b.1 proportion of medium- and high-tech industries in value added, and access to mobile networks. This perpetuates a system in which developing countries are to follow a modernization-theory trajectory.

Fundamentally, there is an abiding, specific understanding of innovation and technology as prerequisites for sustainable development, reliant on innovation to solve resource overuse (Lorek and Spangenberg 2014). The assumption relies on a type of environmentalism, the gospel of eco-efficiency (Anguelovski and Martinez-Alier 2014), which suggests that the material economy can grow without plundering natural resources. This logic argues that technical improvements and substitutions allow absolute decoupling to take place, despite all historical evidence to the contrary (Fletcher and Rammelt 2017; Parrique et al. 2019). Innovation and infrastructure are understood as a technological, patentable or financial question ‘because that is the only kind of innovation upon which a business model can be based’ (de Saille and Medvecky 2016, p. 12). Crucially, these indicators suggest an understanding of technology, infrastructure and sustainability which is firmly rooted in modernity–coloniality (Jiménez and Roberts 2019). This is not to suggest in any way that technology and science do not come from other traditions; however, it is to emphasize that there is a particular understanding of science and technology being advanced by the SDGs.

### Universality in the SDGs

**Fig. 4:**
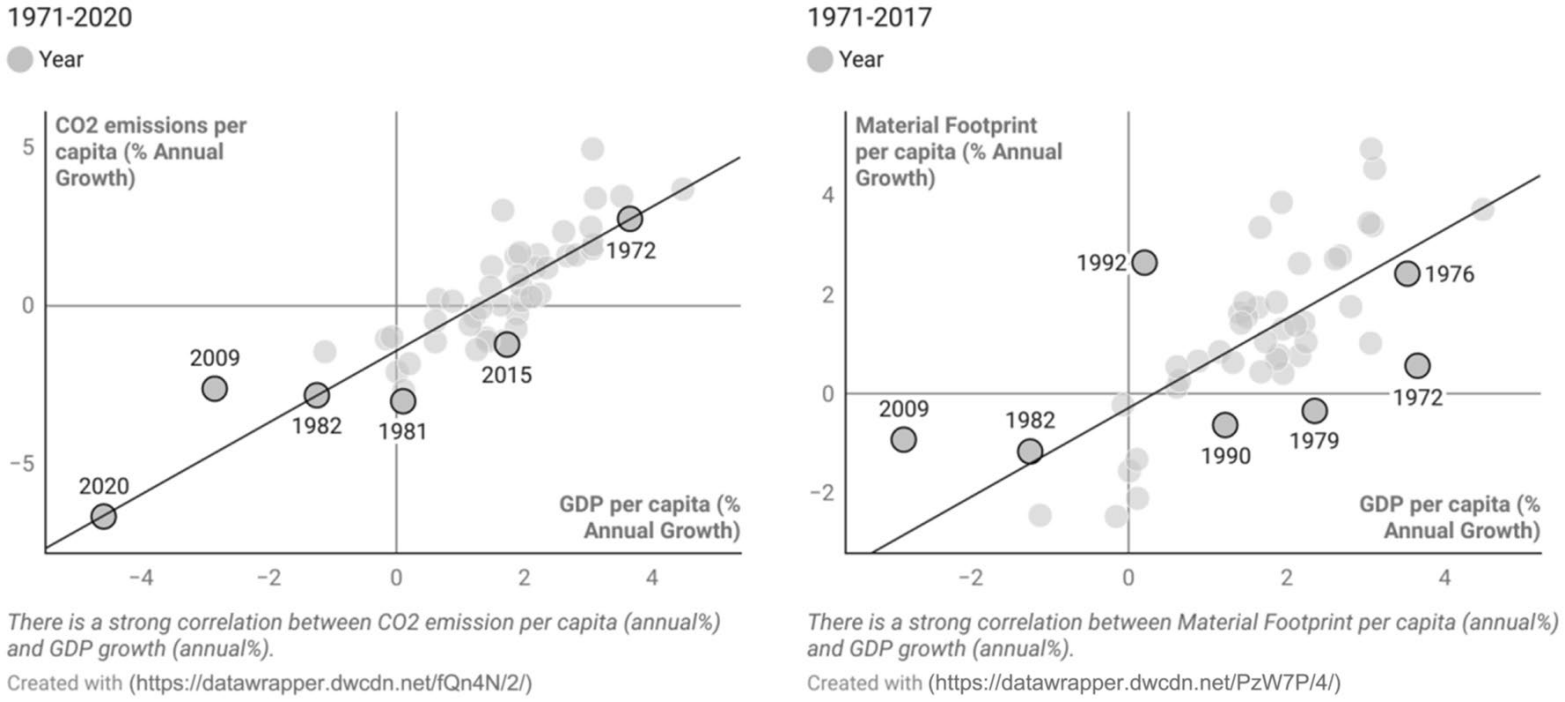
Relationships between GDP rate of growth per capita, material footprint annual growth per capita (1971–2017) and CO_2_ emissions annual growth per capita (1961–2014), all of them being stationary variables. Using non-stationary time series data produces unreliable and spurious results and leads to poor understanding. The main problem is due to GDP per capita and also both footprints per capita being auto-correlated. Auto-correlation refers to the degree of correlation between the values of the same variables across different observations in the data. That is why we have use growth rates of all the included variables. According to the Dicky–Fuller test, the growth rate of GDP and the growth rates of the footprint are stationary variables. Source: Own figure based on United Nations Environment Programme (UNEP) (2020) and World Bank (2020b)

A universal assumption of GDP growth being desirable is visible, e.g., in SDG 8. For the enumerated outcome targets 8.1–8.10, there are seven Tier I indicators, i.e. for which both data and methodology are available. Of them, two refer to GDP growth: GDP percentage growth (8.1.1) and GDP growth per employed person (8.2.1); two others are about access to banks (8.10.1, 8.10.2); two are about unemployment (8.5.2, 8.6.1); and another covers domestic material consumption and material footprint, which are divided by GDP (8.4.2) and by population (8.4.1.). Moreover, targets and indicators particularly among means of implementation targets (e.g. 8.a, 9.c, 15.a, 15.b) actively rely on growth, in terms of resource consumption or funding. However, time-series data at a global level show a positive and significant correlation between GDP annual growth and material footprint annual growth (*r* = 0.72; *p* = 0.00) as well as CO_2_ emissions annual growth (*r* = 0.87, *p* = 0.000) (Brockington et al. 2020) (Fig. 4). Figure 4 shows that the annual change in CO_2_ emissions closely follows the annual change in GDP: the recessions of the mid-1970s and the early 1980s caused major declines in worldwide emissions and the use of materials, with the Great Recession (2009) and coronavirus crisis (2020) also affecting carbon emissions.

Empirically, there are thus strong indications that 8.1 (annual growth rate of GDP per capita) violates the environmental sustainability objectives of the SDGs (Hickel 2019). However, the 8.1.1 indicator of real GDP growth per capita, in contrast to the target’s reference to ‘national circumstance’, does not differentiate between different countries or income levels in terms of who can grow how much, nor does it commit any rich countries to stricter decarbonizing or decoupling targets, neither in relatives nor absolutes. Although there is some attempt in the target to suggest that growth is needed more in least-developed countries, the indicator does not operationalize that distinction, nor does it account for the socio-ecological consequences of growth. Moreover, this universalist definition of economic betterment as GDP growth precludes other ways of imagining prosperity through de-growth or stasis (Brockington et al. 2020). Consequently, the current conceptualization of growth in the SDGs favors privileged populations and their resource-intensive lifestyles, while adding to the question of whether sustainable development is an oxymoron (Latouche 2008) given the socio-ecological consequences of universalizing privileged lifestyles.

**Fig. 5:**
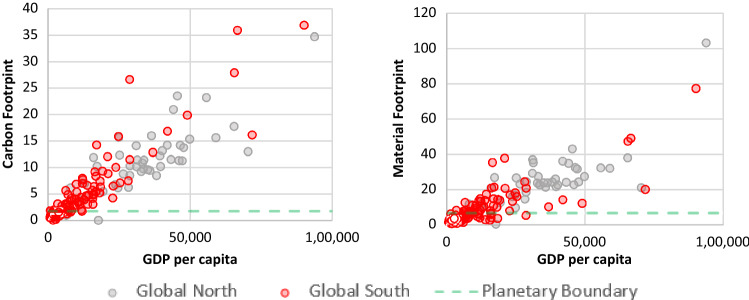
Relationships between GDP per capita (2017) and material footprint per capita and carbon footprint per capita (2018, constant 2011 PPP). Source: Own figure based on United Nations Environment Programme (UNEP) (2020), World Bank (2020b) and EORA-MRIO (2016)

Although Fig. 5, which is based on Hickel’s (2019) per-capita planetary boundary, demonstrates that upper-middle-income and high-income countries acutely need to reduce their material footprint, indicator 8.4.1 creates no such urgency given its lacking focus on absolute levels and who is consuming these resources. Whereas SDG 8.4 (see above for precise wording) recognizes industrialized countries’ outstanding responsibility, the indicator’s framing does not support such analysis, never mind mandate. Secondly, although SDG 8 and SDG 12 use domestic material consumption as an indicator, the scope of domestic material consumption is limited to the materials directly used by any national economy, and does not include upstream raw materials.^16^ In a globalized economy, where rich countries have outsourced much of their production to poorer countries, this side of material consumption has been shifted off their balance sheets (Hickel and Kallis 2019). Despite the evidence shown in Figs. 4 and 5, neither SDG 8 nor SDG 12 operationalize this link between economic growth and environmental degradation, nor make connections to, e.g., SDG 13 or SDG 15 or planetary boundaries.

Universalist ideas equally inform the two environmental SDGs investigated: climate action and life on land. In SDG 13, while one could assume that the omnipresence of Tier II (2) and Tier III (5) indicators suggests an attempt to be radical, the opposite seems to be the case. Process indicators rather than outcome indicators are prevalent, with five indicators counting the number of national or local governments with documents including disaster preparedness plans (13.1.2, 13.2.1, 13.3.1, 13.4.1, 13.1.3). Among them, the only Tier I, i.e. currently measurable, indicator is 13.1.2, on national disaster strategies. This means that progress on climate change is measured in SDG 13 by how many countries have specific documents in place that correspond with generally capitalist, Eurocentric ideas about climate change focusing on technology and finance (Salleh 2016). In terms of equity, neither SDG 13 nor its indicators make explicit that low-income countries and their populations are particularly at risk (Kroll et al. 2019) because many intersecting vulnerabilities will be magnified because of climate change. This will affect non-industrialized countries disproportionately relative to their historical carbon emissions (IPCC 2018). This lack of awareness of privileged lifestyles’ socio-ecological consequences suggests again that, in keeping with the double bind, a nature-protection message is inevitably frustrated by the supposed superior necessity of continuing economic development (Requena and Moreno 2018; Rodríguez Victoriano 2002).

**Fig. 6:**
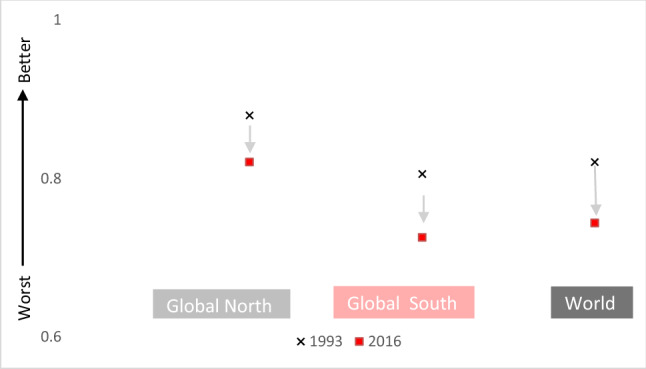
Changes in Red List Index 1993–2016. Source: Own figure based on UN Stats data and Zeng et al. (2020)

**Fig. 7:**
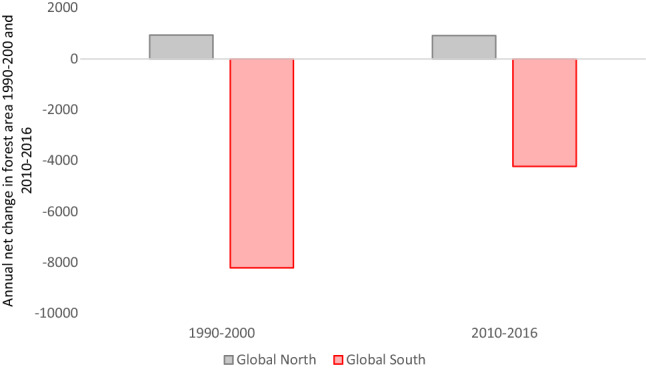
Annual net change in forest area 1990–2000 and 2010–2016. Thousands of hectares. Source: Own figure based on UN stats and Zeng et al. (2020)

These unjust dynamics are equally visible in SDG 15, life on land, and its indicators. A heavy reliance on protected areas in SDG 15 (e.g. 15.1., 15.2., 15.4) recalls the above-explained ‘cult of wilderness’ (Martinez-Alier 2002b). SDG 15’s reliance on protected areas implies that restricting adjacent populations will solve the conservation problem, which also furthers the problematic suggestion of environmental degradation being caused by poverty (Dunlap and York 2008). It does not remedy, and arguably promotes by relying on tourism revenue, significant ecological footprints in the Global North. It is noticeable that, unlike the SDG Dashboards (Sachs et al. 2017, 2018, 2019), there is no indicator for SDG 15 which references international trade or commerce. This inattention to trade and travel neglects to link to significant privileged ecological footprints contributing to biodiversity loss and climate change, thus placing the responsibility for change again on non-industrialized countries. Key SDG 15 indicators, for 15.5 on biodiversity loss (the red list index, Fig. 6) and 15.2 on forests (forest area change, Fig. 7), show privileged countries as succeeding, despite abiding resource-intensive lifestyles. As the below figures show, Global North countries perform better in terms of the red list index and forest area change (in %, 2010–2017). However, there is no connection to SDG 12’s material footprint, despite SDG 12 encompassing three targets which explicitly reference nature or the environment (12.2, 12.4 and 12.8), nor to overall carbon emissions. It is only by ignoring equity questions around resource flows and socio-ecological consequences that the suggestion of universal applicability for these concepts and measurements can be maintained.

Our findings thus confirm Gupta and Vegelin’s (2016) observation that the SDGs do not substantively redefine the growth concept given limited ecospace and a need to enhance human welfare, particularly of the disadvantaged. As currently constituted, the SDGs will not avoid environmental destruction (Zeng et al. 2020). In sum, supposedly universal understandings of economy and ecology place responsibility for change unfairly on the Global South, while ignoring socio-ecological consequences for the vulnerable.

## Concluding observations

This article has investigated five SDGs with two interrelated foci. In answer to our first research question, we used the work of, e.g., Santos (2014), Hall (1992), Connell (2007), Bhambra (2004), Quijano (1992) and Maldonado-Torres (2016) to build a decolonial lens highlighting systematic absences, universality and modernity–coloniality. Despite the SDGs’ audacious aspiration and invitation of diverse voices in its consultation, we retraced in the detail of SDGs 8, 9, 12, 13 and 15 unacknowledged trade-offs or, with Santos (2014), absences, e.g. between continued economic growth for the privileged, and its unacknowledged socio-ecological implications elsewhere. While some SDGs (e.g. 9 or 12) mention the need for innovation or relative decoupling with reference to carbon or natural resources vis-à-vis growth, the SDGs do not connect the respective indicators to each other, nor link to ecological integrity (Eisenmenger et al. 2020) or the crucial question of absolute resource use. Particularly at the indicator level, we found a recurring implicit or explicit reliance on supposedly universal notions and constructs steeped in modernity–coloniality (Bhambra 2004; Quijano 2007) which ultimately serve the Global North (Figs. 3–7): this ranged from understandings of innovation and technology (SDG 9) to lacking commitments to reducing absolute carbon (SDG 13) or biodiversity loss (SDG 15) through more equitable and genuinely sustainable privileged lifestyles (SDGs 8 and 12). In sum, Quijano’s (1992) observation of the global impact of a supposedly universal modernity–coloniality paradigm is arguably confirmed by the very presence of siloed, supposedly universal SDG indicators, with SDG implementation often focused on single goals (Alcamo et al. 2020).

In answer to our second research question, we detailed these dynamics’ significant global-equity implications. We concur with Menton et al.’s (2020) assessment that justice is not at the heart of the SDGs. As currently constituted, the SDGs risk prioritizing the interests of the rich (Gupta and Vegelin 2016). While the SDGs constitute an advance on the Millennium Development Goals by expanding responsibility to the Global South and Global North, our analysis demonstrated how in the SDGs responsibility for transformations is placed unfairly on the Global South, and the socio-ecological consequences of privileged lifestyles particularly for the most vulnerable are hidden. Fundamentally, the SDGs enshrine the primacy of a specific, market-oriented paradigm into another supposedly universal project (Weber 2017, p. 400–401), which risks prioritizing economic growth over social and ecological issues (Gupta and Vegelin 2016) much like the sustainable development agenda (see, e.g., Adams 2010).

Overall, our analysis thus confirms that the SDGs rely on Northern-inspired solutions to environmental degradation such as technology transfer over redefining growth to prioritize planetary boundaries and human welfare (Gupta and Vegelin 2016). What is worse, the reliance on growth and development as solutions particularly for the poorest nations furthers the myth of trickle down (Salleh 2016) in keeping with the Brundtland Report’s logic (Guha and Martinez-Alier 1997). This logic thus perpetuates the supremacy of industrialization and development as universal solutions despite the vast socio-ecological implications of an imperial mode of living by the privileged (Brand and Wissen 2017; Lang and Hoetmer 2018). This is especially unfair for low-income countries which, historically and currently, consume less material per capita, reaffirming an abiding ecological debt or socio-ecology subsidy (Martinez-Alier 2002a; Rice 2009, among others). This subsidy, which began in the colonial era and continues to this day, not only enriches the privileged, but also impoverishes and degrades the land, culture and capacity-building potential especially of non-privileged communities (Rice 2009).

Our analysis has thus confirmed our argument that absences, modernity–coloniality and universality in the SDGs call into question whether the SDGs’ wider objective can be attained, i.e. safeguarding advances for people, planet, prosperity, peace and partnerships. Our original contribution was bringing together sustainable development and decolonial literatures with in-depth analysis of five Sustainable Development Goals. Our article is intended as an initial contribution to a larger conversation. We invite further research, especially on decolonial thought across all 17 SDGs, to test and challenge both our conceptual lens and our findings across all goals. We equally support efforts to revise current SDGs to address the shortcomings we identify such as the rich making murderous demands on planet and people (Illich 1973; Krauss 2021). Equally, we would encourage starting early on building a viable post-2030 agenda which uses insights from our and other studies on the SDGs and alternatives to them (e.g. Hidalgo-Capitán et al. 2019) to accentuate a post-2030 agenda’s transformative potential for genuine equity, sustainability and decoloniality.

The SDGs were established with the objective of going beyond previously agreed language. However, our analysis prompts us to ask to what extent the SDGs’ very existence subverts the change they purport to aspire to: talking about environmental degradation and sustainable consumption suffices to suggest that they are ‘transforming our world’, without actually taking path-altering steps to produce an equitable, sustainable ‘future we want’. On balance, this strongly suggests a performative nature of the SDGs: while the existence and language – the style – of the SDGs suggests change, our closer analysis of goals, targets and indicators suggests that they serve to redirect the eye of the beholder away from abiding unresolved contradictions – the substance. The SDGs, being a global commitment, provide us with an opportunity to rethink how we want to co-exist in this world. To the extent that we recognize the connections (or lack thereof), the dominance of specific, problematic ways of viewing the world, and inequities, we can start to redirect the debate towards a different, more equitable and ultimately more sustainable direction which challenges particularly the Global North’s conduct.

## Appendix 1

See Table 1.

**Table 1:** SDG indicator connections. Source: Authors, based on UN Stats (2020)

Finding	Number of indicators	Percentage of total	Found for SDG indicators
No metadata	11	16.92	1 SDG 8, 4 SDG 12, 6 SDG 13
No connections	33	50.77	9 SDG 8, 9 SDG 9, 10 SDG 15
1 connection	6	9.23	2 SDG 8, 3 SDG 12
2–4 connections	7	10.77	
5+ connections	8	12.31	3 SDG 9
Total	65		

## Appendix 2

See Table 2.

**Table 2:** SDG 8, 9, 12, 13 and 15 indicator connections. Source: Authors, based on UN Stats (2020)

	SDG 8	SDG 9	SDG 12	SDG 13	SDG 15	Total
Total number of SDG × indicators	17	12	14	8	14	65
Total number of SDG × indicators with no metadata	1	0	4	6	0	11
Total number of SDG × indicator connections	21	17	21	24	10	93
Intra-goal connections (within the same SDG)	7	2	11	5	6	31
Intra-study connections (to SDGs within our study)	2	1	2	6	0	11
SDG × indicators with no connections	9 out of 17	9 out of 12	4 out of 14	1 out of 8	10 out of 14	33 out of 65

## Appendix 3

See Table 3.

**Table 3:** List of connections/absence of connections among five SDGs studied. Source: Authors, based on UN Stats (2020)

SDG	Connections	No connections
SDG 8 (growth)	− 1 (no poverty) − 2 (hunger) − 5 (gender equality) − 10 (reducing inequalities) − 16 (peace and security)	− to 9, despite 8.2 and 8.3 reference to innovation − to 13 (climate action), 15 (life on land), despite 8.4 reference to natural resources − 9 of 17 indicators (Appendix 2)
SDG 9 (industry, innovation, infrastructure)	− 1 (poverty) − 2 (hunger) − 3 (health) − 11 (cities) − 12 (production and consumption) − 13 (climate) − 14 (life below water) − 17 (partnerships)	− to 8 − to 13, despite 9.4 on retrofitting infrastructure for resource efficiency being measured by CO_2_ emissions per unit of value added − 9 of 12 indicators, despite high trade-offs identified by Pradhan et al. (2017)
SDG 12 (sustainable consumption and production)	− 3 (good health) − 4 (education) − 8 (growth) − 11 (sustainable cities) − 12 − 14 (life below water) − 11 within SDG 12 − 11 for one target (12.1, 10-year production and consumption framework)	− 4 of 14 no metadata − 13 (climate action), 15 (life on land)
13 (climate action)^a^	− All 24 connections for one indicator: 13.1.3, on proportion of local governments with disaster risk reduction strategies (21 to targets, not indicators, including five within SDG 13) − 1 (poverty) − 2 (hunger) − 3 (health) − 4 (education) − 6 (water) − 9 (innovation) − 11 (sustainable cities) − 14 (life below water) − 15 (life on land) − 10 connections (6 intra-goal)	− 5 out of 8 no metadata − to 8, 9 despite references to resource efficiency and carbon/significant trade-offs with other goals (Lusseau and Mancini 2019; Pradhan et al. 2017) − 10 of 14 indicators
15 (life on land)		
	− 2.4 (production area under sustainable agriculture) − 11.3 (sustainable urbanization) − 6.6 (water-related ecosystems)	− more systematically to 2 on hunger/agriculture (Larson and Larson 2019: connections between environmental performance and reducing hunger) − to 1 (poverty), 4 (education), 10 (reducing inequalities), despite Pradhan et al.’s (2017) trade-offs

^a^We have counted 13.1.2, ‘Number of countries that adopt and implement national disaster risk reduction strategies in line with the Sendai Framework for Disaster Risk Reduction 2015–2030’, as having no metadata, as the 13.1.2 metadata offered on the UN Stats website as of 1 February 2020 does not link to this indicator, but instead links to 13.1.3 in terms of content.

## Footnotes

1 Former permanent representative of Hungary to the UN.

2 Alternative indicators considered by IAEG-SDGs predominantly stemmed from international entities and UN agencies (UN, 2015a).

3 We acknowledge that, in UN Statistics metadata, some of the same indicators are affiliated with multiple, yet different, targets, for example: 8.4.1 and 12.2.1; 8.4.2 and 12.2.2; 15.a.1 and 15.b.1; 15.7.1 and 15.c.1; 1.5.1, 11.5.1, and 13.1.3; 1.5.3, 11.b.2, and 13.1.1.

4 In the ‘related indicators’ sections, some of the references were not to indicators, but targets (e.g. 2.3 rather than 2.3.1). Where the target indicator had only one indicator, we rectified it (i.e. 15.2.1 instead of 15.2); where multiple indicators may be the ‘related’ ones (e.g. 15.1.1 or 15.1.2 for 15.1), we counted the target as stated.

5 The connections are directed, i.e. they start from indicator A and are directed towards indicator B. These connections are not usually reciprocated by the other indicator. Zoomable file available on request.

6 For determining which countries are from the Global South, we used the list of countries included in the Global South Countries in the Finance Centre for South-South Cooperation – an official institution related to the UN. However, we found in some UN reports (see for instance UN 2018b) that Global South countries have been used as a synonym for developing countries – in other words, countries catalogued as developing in the standard country or area codes for statistical use (known as M49). The list of developing countries included in M49 contains seven more countries than the list included in the Finance Centre for South-South Cooperation. These countries are: Armenia, Azerbaijan, Hong Kong, Kyrgyzstan, Mexico, Palau and Uzbekistan.

7 Both carbon emissions and material footprint are divided by 7.509 billion people, as of 2017 (Hickel 2020).

8 Hickel (2020) estimates what the remaining carbon budget for the century was in 2015 if we are to have a 67% chance of staying between 1.5 °C and 2 °C, averages the two budgets for 2018, subtracts emissions since 2015 and divides by the remaining years of the century.

9 Though the UN statistics division/the reporting bodies responsible do not indicate connections between indicators and their affiliated goals (e.g. 8.1.1 and SDG 8), we have added in connections between each goal and the indicators with which they are affiliated for the sake of clarity.

10 9.1: ‘Develop quality, reliable, sustainable and resilient infrastructure, including regional and transborder infrastructure, to support economic development and human well-being, with a focus on affordable and equitable access for all’ (UN 2019b, p. 9).

11 8.4: ‘[i]mprove progressively, through 2030, global resource efficiency in consumption and production and endeavour to decouple economic growth from environmental degradation … with developed countries taking the lead’ (UN 2019b: 8).

12 As Jackson and Victor (2019) claim, decoupling refers to a situation when ‘Economic output becomes progressively less dependent on material throughput. In this way, it is hoped, the economy can continue to grow without breaching ecological limits’ (Jackson and Victor 2019, p. 67). However, it is necessary to distinguish between relative and absolute decoupling. Relative decoupling refers to a decline in the ecological intensity per unit of economic output. In this situation, resource impacts decline relative to the GDP. But they do not necessarily decline in absolute terms. Impacts may still increase, but at a slower pace than growth in GDP. The situation in which resource impacts decline in absolute terms is called absolute decoupling.

13 9: ‘Build resilient infrastructure, promote inclusive and sustainable industrialization and foster innovation’ (UN 2019b, p. 9).

14 9.2: ‘Promote inclusive and sustainable industrialization and, by 2030, significantly raise industry's share of employment and gross domestic product, in line with national circumstances, and double its share in least developed countries’ (UN 2019b, p. 9).

15 We have also explored other contradictions between indicators targeting SDG9 and the analysed footprints. In this sense we have found that countries that have a high expenditure in research and development and a high proportion of medium and high-tech industry value added – mostly countries from the Global North – have also a high material and carbon footprint per capita.

16 Domestic material consumption does not include the upstream raw materials related to imports and exports originating from outside the national economy. Hence, if a car is imported, only its weight is counted, and not all the energy, water and other materials required to produce the metal, rubber and plastic it contains.
